# Synthesis of Chlorophyll-Binding Proteins in a Fully Segregated Δ*ycf54* Strain of the Cyanobacterium *Synechocystis* PCC 6803

**DOI:** 10.3389/fpls.2016.00292

**Published:** 2016-03-17

**Authors:** Sarah Hollingshead, Jana Kopečná, David R. Armstrong, Lenka Bučinská, Philip J. Jackson, Guangyu E. Chen, Mark J. Dickman, Michael P. Williamson, Roman Sobotka, C. Neil Hunter

**Affiliations:** ^1^Department of Molecular Biology and Biotechnology, University of SheffieldSheffield, UK; ^2^Sir William Dunn School of Pathology, University of OxfordOxford, UK; ^3^Institute of Microbiology, Centre Algatech, Academy of Sciences of the Czech RepublicTřeboň, Czech Republic; ^4^Faculty of Science, University of South BohemiaČeské Budějovice, Czech Republic; ^5^ChELSI Institute, Department of Chemical and Biological Engineering, University of SheffieldSheffield, UK

**Keywords:** Ycf54, *Synechocystis* 6803, chlorophyll, photosystem II, protochlorophyllide, Mg-protoporphyrin IX methylester cyclase

## Abstract

In the chlorophyll (Chl) biosynthesis pathway the formation of protochlorophyllide is catalyzed by Mg-protoporphyrin IX methyl ester (MgPME) cyclase. The Ycf54 protein was recently shown to form a complex with another component of the oxidative cyclase, Sll1214 (CycI), and partial inactivation of the *ycf54* gene leads to Chl deficiency in cyanobacteria and plants. The exact function of the Ycf54 is not known, however, and further progress depends on construction and characterization of a mutant cyanobacterial strain with a fully inactivated *ycf54* gene. Here, we report the complete deletion of the *ycf54* gene in the cyanobacterium *Synechocystis* 6803; the resulting Δ*ycf54* strain accumulates huge concentrations of the cyclase substrate MgPME together with another pigment, which we identified using nuclear magnetic resonance as 3-formyl MgPME. The detection of a small amount (~13%) of Chl in the Δ*ycf54* mutant provides clear evidence that the Ycf54 protein is important, but not essential, for activity of the oxidative cyclase. The greatly reduced formation of protochlorophyllide in the Δ*ycf54* strain provided an opportunity to use ^35^S protein labeling combined with 2D electrophoresis to examine the synthesis of all known Chl-binding protein complexes under drastically restricted *de novo* Chl biosynthesis. We show that although the Δ*ycf54* strain synthesizes very limited amounts of photosystem I and the CP47 and CP43 subunits of photosystem II (PSII), the synthesis of PSII D1 and D2 subunits and their assembly into the reaction centre (RCII) assembly intermediate were not affected. Furthermore, the levels of other Chl complexes such as cytochrome *b*_6_*f* and the HliD– Chl synthase remained comparable to wild-type. These data demonstrate that the requirement for *de novo* Chl molecules differs completely for each Chl-binding protein. Chl traffic and recycling in the cyanobacterial cell as well as the function of Ycf54 are discussed.

## Introduction

Chlorophylls (Chl) and Chl binding proteins are essential components of the photosynthetic apparatus. Together they act as principal light harvesting and energy transforming cofactors in photosynthetic organisms, as demonstrated by the structures of both photosystem I (PSI) and photosystem II (PSII; Jordan et al., 2001; Zouni et al., 2001; Umena et al., 2011). It is likely that at least for large Chl-binding subunits of PSI (PsaA, PsaB) and PSII (CP43, CP47) Chl molecules must be inserted into these proteins co-translationally as a prerequisite for correct protein folding (Chua et al., 1976; Eichacker et al., 1996; Müller and Eichacker, 1999; Chidgey et al., 2014). As demonstrated recently using a cyanobacterial Δ*chlL* mutant, which is unable to synthesize Chl in the dark, the availability of *de novo* Chl molecules is ultimately essential for synthesis of all central subunits of both photosystems (Kopečná et al., 2013). Nonetheless, there are unexplained aspects of the assembly of PSII subunits, such as the particular sensitivity of the CP47 subunit to the lack of *de novo* Chl (Dobáková et al., 2009; Kopečná et al., 2015).

The Chls are a group of modified tetrapyrrole molecules distinguished by their fifth isocyclic or E ring, the geranylgeranyl/phytol moiety esterified at C17 and a centrally chelated magnesium ion. The isocyclic ring arises from the cyclisation of the methyl-propionate side-chain at C-13 to the C-15 bridge carbon between rings C and D. In oxygenic phototrophs this biosynthetic reaction is catalyzed by the oxidative Mg-protoporphyrin IX monomethyl ester cyclase (MgPME-cyclase), which incorporates atmospheric oxygen into the C13^1^ carbonyl group (Porra et al., 1996). Although studied in some detail, the enzyme responsible for the aerobic cyclisation reaction remains the least understood in the Chl biosynthesis pathway. The first gene identified as encoding an oxidative cyclase component was the *Rubrivivax gelatinosus acsF* (aerobic cyclisation system iron containing protein) locus (Pinta et al., 2002). Subsequently, AcsF homologs have been identified in all studied oxygenic photosynthetic organisms (Boldareva-Nuianzina et al., 2013).

The Ycf54 protein (12.1 kDa) has been shown recently to interact with the AcsF homolog Sll1214 (hereafter CycI; Peter et al., 2009) in the cyanobacterium *Synechocystis* PCC 6803 (hereafter *Synechocystis*; Hollingshead et al., 2012). Demonstrations that partial elimination of Ycf54 strongly impairs the formation of PChlide and causes Chl deficiency in both cyanobacteria and plants (Albus et al., 2012; Hollingshead et al., 2012) led to speculations that this protein is a catalytic subunit of the MgPME cyclase (Bollivar et al., 2014). Here, we clarify this issue by achieving the complete deletion of the *ycf54* gene in *Synechocystis*. Although the Chl content in this strain was very low, the MgPME-cyclase was apparently active, which demonstrated that the Ycf54 protein is not an essential subunit of the MgPME cyclase. On the other hand, the mutant contained a very low level of CycI and lacked a high-mass complex associated with the light-dependent PChlide oxidoreductase enzyme (POR). The greatly limited formation of PChlide in the *ycf54* mutant provided an opportunity to assess the sensitivity of assembly pathways for all known Chl-proteins in cyanobacteria to the availability of *de novo* Chl. Interestingly, the deletion of the *ycf54* gene almost abolished the synthesis of PsaA/B subunits and PSII antennas CP47 and CP43, whereas the accumulation of other Chl-proteins showed little or no defects.

## Experimental Procedures

### Growth Conditions

*Synechocystis* strains were grown photomixotrophically in a rotary shaker under low light conditions (5 μmol photons m^-2^ s^-1^) at 30°C in liquid BG11 medium (Rippka et al., 1979) supplemented with 10 mM TES-KOH (pH 8.2) and 5 mM glucose.

### Construction of the Δ*ycf54 Synechocystis* Strain

In order to disrupt open reading frame *slr1780* (*ycf54*), we prepared a construct for replacing the most this gene (bp 64–276) by a Zeocin resistance cassette. The sequences up- and down-stream (~300 bp) of the *ycf54* gene were amplified with the relevant primers and fusion PCR in conjunction with megaprimers (Ke and Madison, 1997) were used to anneal these either side of the Zeocin resistance cassette. The resulting PCR product was transformed into the GT-W *Synechocystis* substrain (Bečková et al., submitted) and transformants were selected on a BG11 agar plate containing 2 μg ml^-1^ Zeocin. Complete segregation was achieved by sequentially doubling the concentration of antibiotic to a final concentration of 32 μg ml^-1^ Zeocin.

### Cell Absorption Spectra and Determination of Chl Content

Absorption spectra of whole cells were measured at room temperature using a Shimadzu UV-3000 spectrophotometer (Kyoto, Japan). To determine Chl levels, pigments were extracted from cell pellets (2 ml, OD_750_ = ~0.5) with 100% methanol and the Chl concentration was determined spectroscopically (Porra et al., 1989).

### Analysis of Pigments by HPLC

Pigments were extracted from equal quantities of cells by the method described in Canniffe et al. (2013) and separated on a Phenomenex Aqua C_18_ reverse phase column (5 μM particle size, 125 Å pore size, 250 mm × 4.6 mm) at a flow rate of 1 ml min^-1^. 3-formyl-MgPME was purified on a Fortis Universil C_18_ reverse phase column (5 μM particle size, 125 Å pore size, 150 mm × 10 mm) at a flow rate of 3.5 ml min^-1^. Reverse phase columns were run using a method modified from Sobotka et al. (2011). Solvents A and B were 350 mM ammonium acetate pH 6.9/30% methanol (v/v) and 100% methanol, respectively. Pigments were eluted over a linear gradient of 65 to 75% buffer A over 35 min.

### Purification of 3-Formyl-MgPME for NMR Analysis

Pigments were extracted by phase partitioning from 6 L of Δ*ycf54* culture grown to an OD_750 nm_ 1.2. One volume of diethyl ether was added to two volumes of cell culture in a separation funnel and the diethyl ether phase containing 3-formyl-MgPME was separated from the cell culture. Pigments were extracted from the cell culture three times. The diethyl ether was removed by rotary evaporation and the extracted pigments were re-suspended in a small volume of HPLC grade methanol. After centrifugation at 15,000 ×*g* for 10 min, 3-formyl-MgPME was purified by preparative HPLC. Ammonium acetate was removed from the HPLC purified 3-formyl-MgPME by solid-phase extraction on DSC-18 reverse-phase columns (Supelco). Solvents C, D, and E were QH_2_O, 50% methanol (v/v) and 100% methanol, respectively. After equilibration of the column with 1.0 ml solvent D, the purified 3-formyl-MgPME, diluted 1/3 with QH_2_O, was loaded and allowed to enter the column by gravity flow. The column was washed with 1 ml solvent C, then 1 ml solvent D to remove the ammonium acetate. The pigment was eluted into a glass vial with 300 μl methanol. The purified pigment was completely dried in a vacuum centrifuge and stored at –20°C.

### NMR Assignment of 3-Formyl-MgPME

The dried pigment from HPLC was re-suspended in 500 μl methanol-d4 (Sigma), centrifuged to remove any insoluble pigment, transferred to a 5 mm NMR tube and sealed. All NMR experiments were carried out on a Bruker Avance DRX 600 instrument equipped with a cryoprobe at an acquisition temperature of 298 K.

The one-dimensional selective Nuclear Overhauser Enhancement (NOE) experiments were recorded using a double pulsed field gradient spin echo selective NOE experiment (Stott et al., 1995) using an 80 ms 180° Gaussian pulse for the selective excitation and a 1 s mixing time, acquiring 1024 transients at each saturation frequency. The Total Correlation Spectroscopy (TOCSY) experiment was recorded using a 45 ms spin lock at a power of 8.3 kHz. Two carbon Heteronuclear Single Quantum Correlation (HSQC) experiments were recorded with carbon offsets of 60 and 140 ppm.

### 2D Electrophoresis, Immunodetection, and Protein Radiolabeling

Membrane and soluble protein fractions were isolated from 50 ml of cells at OD_750 nm_ ~0.4 according to Dobáková et al. (2009) using buffer A (25 mM MES/NaOH, pH 6.5, 5 mM CaCl_2_, 10 mM MgCl_2_, 20% glycerol). Isolated membrane complexes (0.25 mg/ml Chl) were solubilized in buffer A containing 1% *n*-dodecyl-β-D-maltoside.

To assess protein levels by immunodetection, the protein content of *Synechocystis* lysates was quantified spectroscopically (Kalb and Bernlohr, 1977), separated by SDS-PAGE (Novagen) and transferred to a nitrocellulose membrane. The membranes were probed with specific primary antibodies and then with secondary antibodies conjugated to horseradish peroxidise (Sigma). The primary antibodies used in this study were raised in rabbits as described in Hollingshead et al. (2012), with the exception of CHL27 (anti-CycI), which was purchased from Agrisera (Sweden).

Two-dimensional clear-native electrophoresis was performed essentially as described in Kopečná et al. (2013). Proteins separated in the gel were stained either by Coomassie Blue, or Sypro Orange, followed by transfer onto a PVDF membrane. Membranes were incubated with specific primary antibodies, and then with a secondary antibody conjugated with horseradish peroxidase (Sigma).

Radioactive pulse labeling of the proteins in cells was performed using a mixture of [^35^S]Met and [^35^S]Cys (Translabel; MP Biochemicals). After 30 min incubation of cells with labeled amino-acids, the solubilized membranes isolated from radiolabelled cells were separated by 2D-electrophoresis. The stained 2D gel was finally exposed to a phosphor-imager plate, which was scanned by Storm (GE Healthcare) to visualize labeled protein spots.

### Relative Quantification of FLAG-CycI and Captured Proteins in Pulldown Assays

Pulldown assays using N-terminal FLAG-tagged CycI as bait, with both wild-type (WT) and Δ*ycf54* backgrounds, were carried out according to Hollingshead et al. (2012). FLAG eluates were concentrated to 100 μl using Amicon Ultra 0.5 ml 3 kDa MWCO ultrafiltration devices (Millipore). The proteins were then precipitated, reduced and *S*-alkylated according to Zhang et al. (2015). Proteolytic digestion was carried out with 1:25 w/w (enzyme:substrate) pre-mixed trypsin/Lys-C (1 μg/μL, Promega, mass spectrometry grade) at 37°C for 2 h. The samples were then diluted with 75 μl 100 mM Tris-HCl, pH 8.5, 10 mM CaCl_2_ and the digestion allowed to proceed for a further 18 h at 37°C. After the addition of 5μl 10% TFA, the samples were desalted using C_18_ spin columns (Thermo Fisher) and analyzed by nano-flow liquid chromatography (Ultimate 3000 RSLCnano system, Dionex) coupled to a mass spectrometer (Maxis UHR-TOF, Bruker or Q Exactive HF Orbitrap, Thermo Scientific). For Maxis data, mass spectra were internally calibrated with the lock-mass ion at m/z 1221.9906 then converted to MGF format using a script provided by Bruker. Q Exactive data-files were converted to MGFs using MSConvert^1^. Protein identification was carried out by searching against the *Synechocystis* PCC 6803 proteome database (release date 02-08-2015, 3507 entries^2^ using Mascot Daemon v. 2.5.1 running with Mascot Server v. 2.5 (Matrix Science), specifying trypsin as the enzyme in the search parameters and allowing for one missed cleavage. *S*-carbamidomethyl-cysteine and methionine oxidation were selected as fixed and variable modifications, respectively. MS and MS/MS tolerances were set to 0.01 Da and false discovery rates determined by searching of a decoy database composed of reversed protein sequences. The data-files and search results have been uploaded to the ProteomeXchange Consortium^3^ via the PRIDE partner repository (identifier DOI 10.6019/PXD003149).

### Electron Microscopy

Wild-type and Δ*ycf54* cells were harvested in the log phase by centrifugation. Cell pellets were loaded into 200 μm deep specimen carriers (Leica Microsystems), pre-treated with 1% lecithin in chloroform and cryo-immobilized by high-pressure freezing using EM PACT2 (Leica Microsystems). Freeze-substitution was carried out as described by van de Meene et al. (2006) using an automatic freeze substitution unit (EM ASF, Leica). Samples were then infiltrated with graded series (1:2, 1:1, 2:1) of Spurr-acetone mixture (6–8 h for each), twice with 100% Spurr’s resin (SPI Supplies) and finally embedded in fresh resin. The polymerization was performed at 60°C for 48 h. Ultra-thin 70 nm sections were cut on ultramicrotome (UCT, Leica), collected on formvar-coated copper grids and stained with uranyl acetate (5 min) and lead citrate (3 min). Grids were viewed with a JEOL 1010 transmission electron microscope operating at 80 kV equipped with a Mega View III camera (SIS GmbH).

## Results

### Ycf54 Is Not Essential for Activity of MgPME Cyclase

**FIGURE 1:**
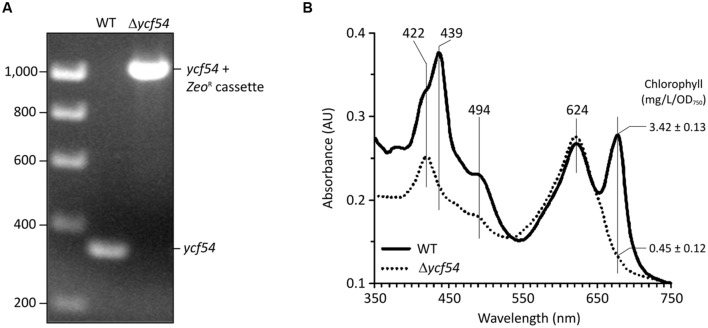
**Deletion of the *ycf54* gene in *Synechocystis* and the whole-cell spectra the resulting Δ*ycf54* strain. (A)** PCR amplification of the *slr1780* (*ycf54*) region to confirm full segregation of the Zeocin resistance cassette. WT, wild-type. **(B)** Whole cell absorbance spectra of *Synechocystis* whole cells grown photomixotrophically under low light conditions. Chl *a* is represented by the 682-nm peak, and phycobiliproteins are represented by the 625-nm peak. Spectra were measured with cells at a similar optical density (OD_750 nm_ = ~0.3) and normalized to light scattering at 750 nm.

In our previous report (Hollingshead et al., 2012) we described a *ycf54*^-^ *Synechocystis* strain harboring an insertion of the Erythromycin resistance cassette in the *ycf54* gene. Although prolonged attempts to fully segregate the mutant allele into all copies of the chromosome were unsuccessful, the phenotype of the partially segregated strain was informative nevertheless, and it exhibited an obvious defect in PChlide formation. However, the capability of *Synechocystis* cells to tolerate deletions of important genes also depends on the ‘WT’ substrain used. For instance, a previous attempt to inactivate *gun4*, another gene crucial for Chl biosynthesis, was achieved in the non-motile *Synechocystis* GT-P substrain but it failed in the motile PCC-M (compare Wilde et al., 2004; Sobotka et al., 2008). Thus, in order to obtain a fully segregated *ycf54* mutant, we prepared a new construct for replacement of the *ycf54* gene and transformed GT-P, GT-S, GT-W and PCC-M substrains; the GT-P substrain has been used in our previous work (Hollingshead et al., 2012). Interestingly, the *ycf54* deletion readily segregated in the GT-W substrain (**Figure 1A**) under low light (5 μmol photons.m^-2^.s^-1^) and photomixotrophic conditions; all attempts to segregate the *ycf54* deletion in other substrains failed (not shown). For the purposes of the work reported here the GT-W substrain is designated as the WT; a detailed analysis of GT-P and GT-W including genome sequencing is presented elsewhere in this issue (Bečková et al., submitted).

The fully segregated Δ*ycf54* strain did not grow photoautotrophically, although supplementation of the growth medium with glucose made photomixotrophic growth possible at light intensities up to 100 μmol photons.m^-2^.s^-1^. Examination of the absorption spectra from cells normalized for optical density at 750 nm (OD_750_), shows that the Chl absorbance maxima at 439 and 679 nm and the carotenoid absorbance maximum at 494 nm are severely depleted in Δ*ycf54*, whilst the absorbance maximum of the phycobiliproteins at 624 nm remains unchanged when compared to the WT (**Figure 1B**). Mutant cells contained only about 13% of WT Chl (**Figure 1B**) and the whole cell spectrum showed a large absorbance peak at 422 nm, indicating a substantial accumulation of MgPME (**Figure 1B**) (Hollingshead et al., 2012).

### Identification of the Chl Precursors that Accumulate in the Δ*ycf54* Mutant

**FIGURE 2:**
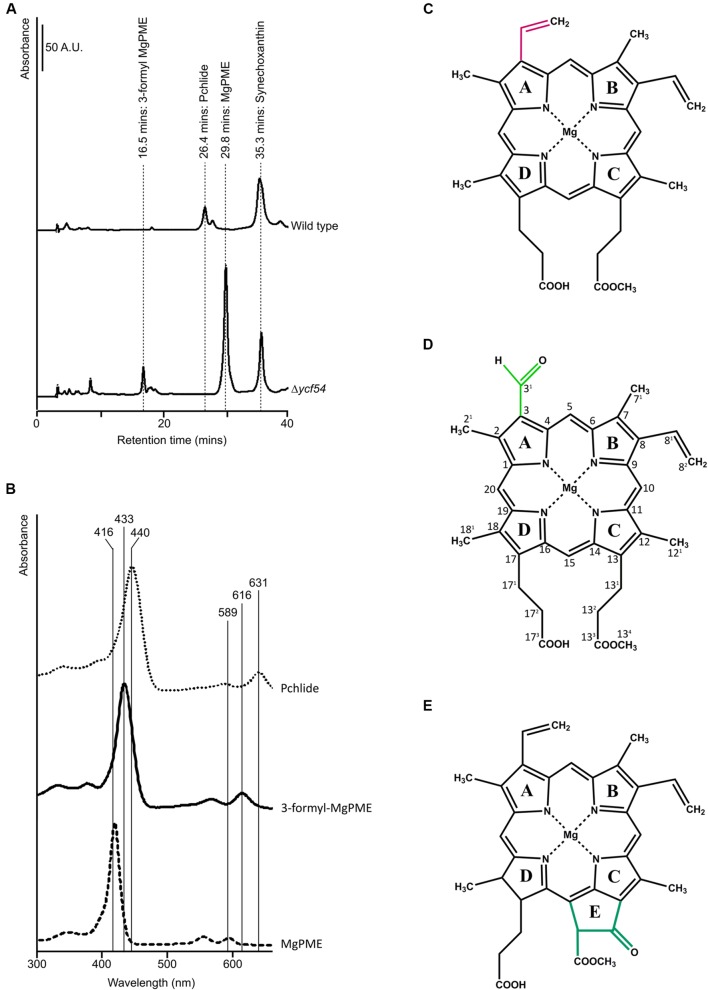
**HPLC analyses of pigments from WT and Δ*ycf54* cells. (A)** Polar pigments were extracted with 80% methanol containing 0.2% (v/v) ammonium from an equal volume of cells at an OD_750 nm_ = ~0.7 and analyzed on a Phenomenex C_18_ column. Separation of precursors was detected by a diode array detector set to 432 nm, the Soret peak of 3-formyl MgPME, which is observed in Δ*ycf54* cells. The elution times of the pigments of interest are indicated. **(B)** Absorbance spectra of MgPME, PChlide and 3-formyl-MgPME, **(C)** Mg-protoporphyrin IX monomethyl ester (MgPME), **(D)** Mg-3-formyl-protoporphyrin IX monomethyl ester (3-formyl-MgPME), **(E)** protochlorophyllide (PChlide).

Previously we reported that Chl biosynthesis in a partially segregated *ycf54^-^* mutant was blocked at the MgPME cyclase step, which causes accumulation of high levels of MgPME, the substrate of the cyclase, and lesser levels of an unknown pigment with a Soret peak at 433 nm (Hollingshead et al., 2012). To examine the photosynthetic precursor pigments present in Δ*ycf54*, methanol extracts from low light grown cells were separated by HPLC (**Figure 2A**). As with *ycf54*^-^, Δ*ycf54* synthesized high levels of MgPME and lesser levels of the unknown pigment. Given that the Soret band of this pigment is situated between the Soret peaks of MgPME at 416 nm and PChlide at 440 nm (**Figure 2B**) we proposed that it could be an intermediate of the cyclase reaction.

**FIGURE 3:**
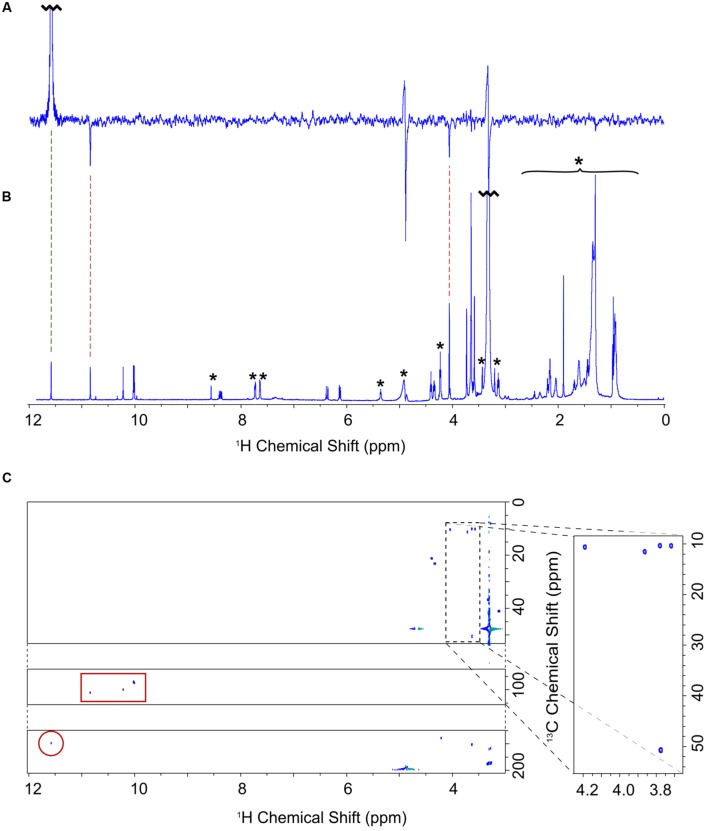
**NMR assignment of the A432 pigment accumulating in the Δ*ycf54* strain. (A)** One dimensional selective NOE spectrum of 3-formyl-MgPME, selectively pulsed at 11.60 ppm (selectively exciting proton 3^1^). Negative signals indicate NOE cross signal with protons 5 and 2^1^ at 10.86 and 4.07 ppm, respectively. **(B)** ^1^H-NMR spectrum of 3-formyl-MgPME. Signals marked with an asterisk are either solvent signals (methanol, water) or impurities (e.g., column matrix). **(C)** ^13^C-HSQC spectrum for 3-formyl-MgPME. The carbon axis is split for clarity. The dashed box indicates methyl signals, expanded on the right. The red box indicates signals from meso protons. The red circle indicates a 3^1^ aldehyde signal.

Nuclear magnetic resonance spectroscopy was used to determine the identity and structure of this unknown pigment, which was extracted by diethyl ether/water phase partitioning from the medium of Δ*ycf54* cells grown under very low light conditions. This pigment was purified to homogeneity by preparative HPLC. The one-dimensional ^1^H spectrum (**Figure 3A**) shows the reasonable degree of purity of the pigment, with impurities indicated by an asterisk; the signals downfield of 5 ppm represent minor contaminants and methanol, whilst the impurity signals upfield of 5 ppm represent solvents, including water. Signals from the unknown pigment were assigned using a combination of ^1^H TOCSY, gradient-selected 1D NOE, and natural abundance ^13^C HSQC spectra.

NOE experiments were carried out with selective saturation of all proton peaks downfield from 3 ppm in order to identify protons with through-space correlation (**Figure 3A**). To cover the full range of ^13^C shifts, two ^13^C HSQC spectra were run with ^13^C offsets of 60 ppm and 140 ppm. Many of the signals have ^1^H and ^13^C chemical shifts similar in frequency to those from MgPME (**Figure 3B**), with the expected TOCSY and NOE connectivities, and can therefore be assigned straightforwardly. The ^13^C HSQC spectra (**Figure 3C**) confirmed the assignments of the four meso protons and the five methyl groups (with the four imidazole methyls having ^13^C shifts of around 10 ppm and the propionate methyl having a shift of 50 ppm). The signals from the 3-vinyl protons were absent, but there is a new signal with ^1^H and ^13^C shifts of 11.6 and 190 ppm, respectively, which can only come from an aldehyde. This signal has NOEs to both the 5-meso and 2-methyl protons, both of which were shifted downfield, and no through-bond connectivity in the TOCSY, verifying that this was a 3-formyl group which had replaced the 3-vinyl group of the MgPME. The NMR data are compiled, together with details of the acquisition parameters, in Supplementary Table 1, including Supplementary Figures S1–S3. Further confirmation that this signal represents a 3-formyl group comes from the ^1^H-NMR spectra of Chl *d* (Fukusumi et al., 2012), which has a clear signal at ~11.5 assigned as the 3-formyl group. Thus, the unknown pigment is magnesium 3-formyl-protoporphyrin IX monomethyl ester (**Figure 2D**).

### Effects of Removal of Ycf54 on Other Chl Biosynthesis Enzymes

**FIGURE 4:**
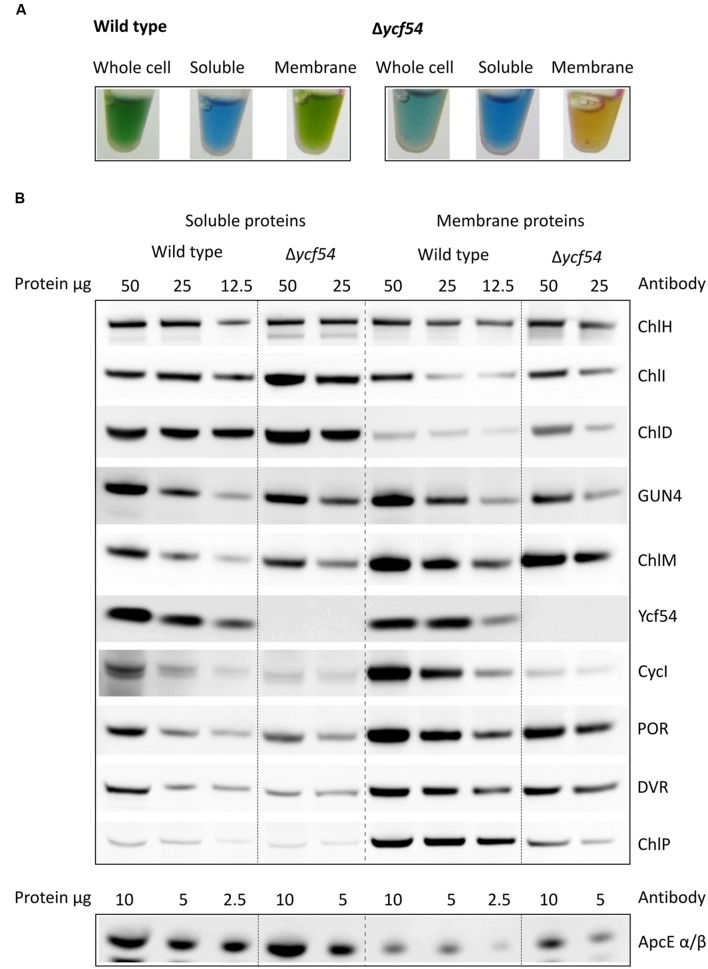
**Levels of Chl biosynthesis enzymes in WT and Δ*ycf54* cells. (A)** Photographs of the whole cell, soluble and solubilized membrane lysate fractions, illustrating the changes in pigmentation between WT and Δ*ycf54*. **(B)** Western blot analysis of the soluble and membrane fractions from WT and Δ*ycf54 Synechocystis* strains. Samples of known protein concentration were separated by SDS-electrophoresis and transferred to a nitrocellulose membrane, which was probed with antibodies to the magnesium chelatase subunits (ChlH, ChlI, ChlD, and GUN4), CycI (CHL27), Mg-protoporphyrin IX methyltransferase (ChlM), Ycf54, PChlide oxidoreductase (POR), 3,8-divinyl (proto)chorophyllide reductase (DVR), geranylgeranyl reductase (ChlP). Detection of phycobiliprotein ApcE α/β served as a loading control.

In order to investigate levels of Chl biosynthetic enzymes, and to verify the loss of Ycf54 in the Δ*ycf54* mutant, lysates from WT and Δ*ycf54* cells were fractionated into membrane and soluble components. The appearance of the cell lysate fractions (**Figure 4A**) reflects their pigment composition; the WT whole cell lysate and membrane fractions are green, and in Δ*ycf54* whole cell lysate and membrane fractions are blue and orange, respectively, because of the near-absence of Chl. A western blot of each of these extracts was probed with antibodies raised against a wide range of Chl biosynthesis enzymes (**Figure 4B**). The immunoblot probed with the antibody to Ycf54 shows this protein is distributed evenly between the soluble and insoluble fractions and is not detected in Δ*ycf54*, confirming the full segregation of this mutant. The absence of Ycf54 is also accompanied by a decrease in CycI and geranylgeranyl reductase (ChlP) and increased relative levels of the Mg-chelatase subunits ChlI and ChlD, although no change was detected in the levels of ChlH (**Figure 4B**).

**FIGURE 5:**
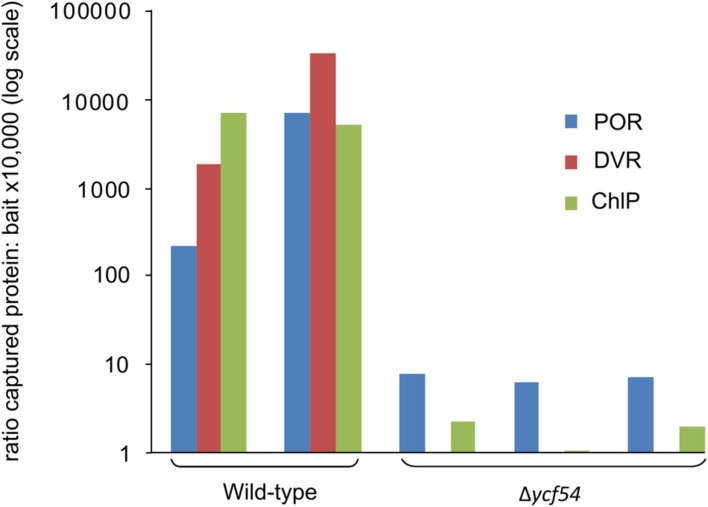
**Relative quantification of CycI, POR, DVR, and ChlP in FLAG-CycI pulldowns against WT and Δ*ycf54* backgrounds, using mass spectrometry.** The bait and captured proteins in FLAG-CycI elutes were identified by mass spectrometry and database searching as described in Materials and Methods. For each analysis, a representative tryptic peptide ion was extracted from the full scan spectra as follows: CycI, AILEEFR, m/z 439.24, 2+; POR, VADELGFPK, m/z 488.26, 2+; DVR (Slr1923), VNKPTLSPNLSVLEEVEK, 666.03 3+; ChlP, AGIETYLFER, 599.81, 2+.

Mass spectrometry was used to quantify the effects of *ycf54* deletion, in terms of the ability of CycI to associate with partner proteins *in vivo*. Pulldown assays with FLAG-tagged CycI are already known to retrieve Ycf54 from cell extracts (Hollingshead et al., 2012), so this experiment was repeated using FLAG- CycI in a Δ*ycf54* background. The amounts of PChlide oxidoreductase (POR), 3,8-divinyl (proto)chorophyllide reductase (DVR) and ChlP captured in pulldown assays by FLAG- CycI/WT and FLAG-CycI in Δ*ycf54* were compared by mass spectrometry. Proteins extracted from FLAG eluates were digested with a combination of endoproteinase LysC and trypsin and the peptide fragments analyzed by nanoLC-MS/MS. The captured proteins were quantified relative to the CycI bait as shown in **Figure 5**. Captured POR levels had decreased significantly in the Δ*ycf54* strain while DVR was reduced to an undetectable level. ChlP was only just detectable in one Δ*ycf54* replicate and relative to CycI by three orders of magnitude in the other two.

### Lack of PChlide Impairs Synthesis of PsaA/B and Inner PSII Antennae but the Accumulation of Other Chl-Binding Proteins Is Not Affected

**FIGURE 6:**
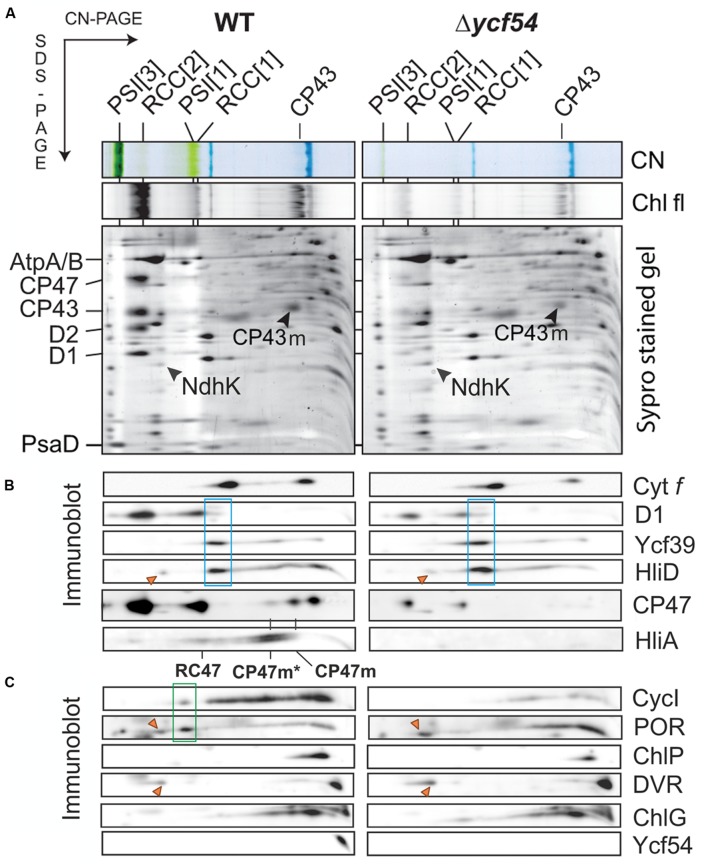
**2D gel-electrophoresis of membrane complexes isolated from WT and Δ*ycf54* cells, followed by immunodetection of PSII assembly complexes and enzymes involved in Chl biosynthesis. (A)** Membrane proteins were separated by 4–14% CN-PAGE, and then in the second dimension by 12–20% SDS-PAGE. The loading corresponds to the same number of cells. The SDS gel was stained by Sypro Orange, blotted and the cytochrome *f* visualized by heme *f* peroxidase activity. Chl fluorescence emitted by PSII and by unassembled CP43 (Chl fl) was detected by Fuji LAS 4000 after excitation by blue light. Designation of complexes is: PSI[3] and PSI[1], trimeric and monomeric PSI, respectively; RCC[2] and RCC[1], dimeric and monomeric PSII core complexes, respectively. **(B)** Immunodetection of PSII assembly complexes. RCII* complex (boxed in blue) was detected using D1, Ycf39, and HliD antibodies. The positions of the CP47 assembly module CP47m, and CP47m associated with High-Light-Inducible proteins HliA/B (CP47m*), are indicated. The HliA signal also marks the position of the PSII core complex lacking CP43 (RC47). **(C)** Immunodetection of Ycf54 proteins and enzymes involved in Chl biosynthesis in the membrane fraction separated by 2D electrophoresis. Highlighted by the green box is a putative high-mass complex (~400 kDa) containing CycI and POR; this complex is not detectable in the mutant. Orange triangles indicate unspecific cross-reactions with a subunit of the NDH complex. ChlG is Chl-synthase; other enzymes are designated as in **Figure 2**.

To evaluate the effects of greatly reduced Chl on the photosystems in Δ*ycf54* compared to the WT, photosynthetic membranes isolated from an equal biomass were gently solubilized with β-DDM and the membrane complexes were resolved by clear native electrophoresis (CN-PAGE), followed by SDS-PAGE in the second dimension. The resulting 2D CN/SDS-PAGE (**Figure 6A**) showed that Δ*ycf54* has drastically reduced levels of both photosystems, whilst the levels of other abundant membrane complexes such as ATP synthase, NADH:ubiquinone oxidoreductase and the cytochrome *b*_6_*f* complex (shown by the western blot) are comparable between the two strains. Interestingly, although the fully assembled PSII complexes in the mutant were barely detectable, this strain still accumulated relatively high levels of unassembled CP43 (**Figure 6A**). This observation suggests a block in formation of the early PSII assembly intermediates, which precedes attachment of the CP43 module and finalization of PSII reaction center core assembly (Komenda et al., 2004).

PSII assembly occurs in a stepwise fashion from four preassembled modules. These consist of one large Chl binding subunit (D1, D2, CP47, or CP43) in addition to several low molecular mass membrane polypeptides, bound pigments and other co-factors (Komenda et al., 2012). Assembly is initiated via the association of D1 and D2 to form the intermediate complex RCII* Knoppová et al. (2014), next the CP47 assembly module is attached, forming RC47, and finally mature PSII is formed by addition of the CP43 module (Boehm et al., 2011, 2012), attachment of the lumenal extrinsic proteins, and light-driven assembly of the oxygen-evolving Mn_4_CaO_5_ complex (Komenda et al., 2008; Nixon et al., 2010). To further investigate the perturbations in PSII assembly, the levels of individual PSII assembly sub-complexes were ascertained by 2D gel electrophoresis and immunodetection (**Figure 6B**). To assess accumulation of the RCII* complex, the immunoblot was probed with antibodies raised against the RCII* components D1, Ycf39, and HliD (Knoppová et al. (2014). **Figure 6B** shows that the level of RCII* is unaffected by the large reduction in cellular Chl levels in the Δ*ycf54* mutant. Next, we investigated if PSII maturation was blocked at CP47 attachment and formation of RC47, by probing the blots with antibodies raised against HliA, a specific component of the CP47 assembly module (Promnares et al., 2006). We found that HliA, and hence the CP47 assembly module, was readily detectable in WT, but could not be detected in Δ*ycf54* (**Figure 6B**), indicating that low Chl abundance in Δ*ycf54* is impairing accumulation of the CP47 assembly module.

Our FLAG-pulldown experiments show that the interactions between CycI, POR, and DVR are significantly reduced in the Δ*ycf54* strain (**Figure 5**), therefore we compared the co-migration of these enzymes on a 2D gel (**Figure 6C**). Evident in the WT is a putative high-mass complex of ~400 kDa (highlighted by the green box), which contains both CycI and POR; this complex was not detectable in the mutant (**Figure 6C**). Interestingly, our 2D gel shows that levels of Chl synthase, ChlG, HliD, and Ycf39, components of a chlorophyll biosynthetic/membrane insertase assembly complex (Chidgey et al., 2014), are unaffected in the Δ*ycf54* mutant (**Figure 6C**).

**FIGURE 7:**
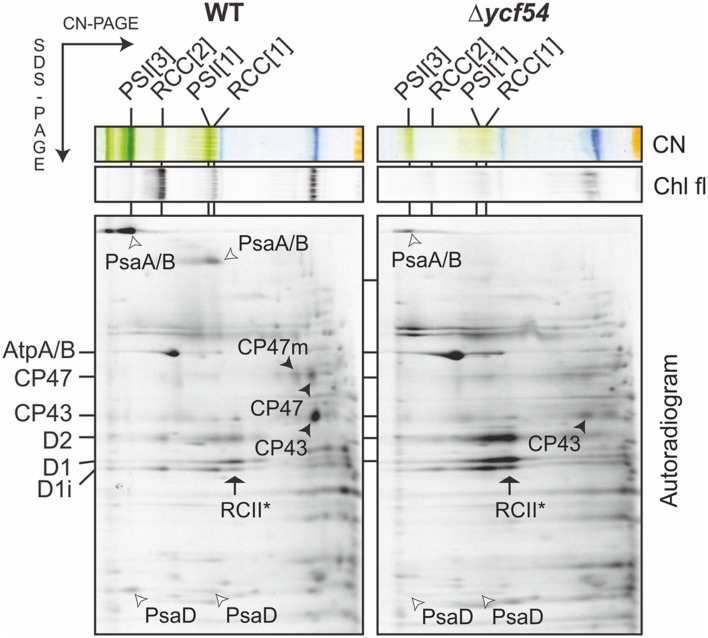
**Synthesis of the Chl-binding proteins in the Δ*ycf54* strain.** WT and mutant cells were radiolabeled with [^35^S]Met/Cys mixture using a 30-min pulse. Isolated membrane proteins were separated by CN-PAGE on a 4–14% linear gradient gel, and another 12–20% SDS-electrophoresis was used for the second dimension. For Δ*ycf54* membrane proteins three-times more cells were loaded than for the control to obtain a detectable signal for weakly labeled proteins (PsaA/B). The 2D gels were stained with Coomassie Blue (the stained gel is shown as Supplementary Figure S4) then dried, and the labeled proteins were then detected by a phosphorimager (Autorad). Protein complexes are designated as in **Figure 6**.

To understand the flux of photosystem biogenesis, we used ^35^S pulse radio-labeling coupled with 2D CN/SDS-PAGE (**Figure 7**; a Coomassie stained gel is provided as Supplementary Figure S4), to compare the levels of protein synthesis between the WT and Δ*ycf54* mutant. As demonstrated in **Figure 7**, the ability of Δ*ycf54* to synthesize the Chl-binding PSI subunits PsaA/B is limited and synthesis of CP47 and CP43 subunits is hardly detectable even though 3-times more Δ*ycf54* protein was loaded onto the gel (See Supplementary Figure S4 for overexposed signal of the CP47). In contrast, there were comparable levels of synthesis of the PSII reaction center core subunits D1 and D2 in the WT and Δ*ycf54* strains. This observation, coupled with the data from our 2D-immunoblot (**Figure 6B**), shows that the D1 and D2 subunits are rapidly assembled into RCII* in Δ*ycf54*, but given the lack of assembled PSII complexes, these RCII* are presumably rapidly degraded in the mutant. Interestingly, in the mutant the unassembled CP43 was still detectable on the stained gel, which contrasted to virtually zero level of unassembled CP47 (**Figures 6A,B** and **7**; Supplementary Figure S4). This observation indicates that both synthesis and stability of the CP47 are impaired in the mutant, whereas the structurally similar CP43 antenna can still accumulate though the synthesis is also very weak (Supplementary Figure S4). Taken together, our data suggest that the depleted levels of *de novo* Chl in Δ*ycf54* specifically hinder the synthesis of PSI and the inner antennae of the PSII. However, given different stability of CP47 and CP43, it is the lack of CP47 protein that blocks assembly of RC47 and thus PSII maturation, sensitizing the PSII assembly pathway to the availability of *de novo* Chl.

### Lack of *De Novo* Chl Affects Ultrastructure of Δ*ycf54* Cells

**FIGURE 8:**
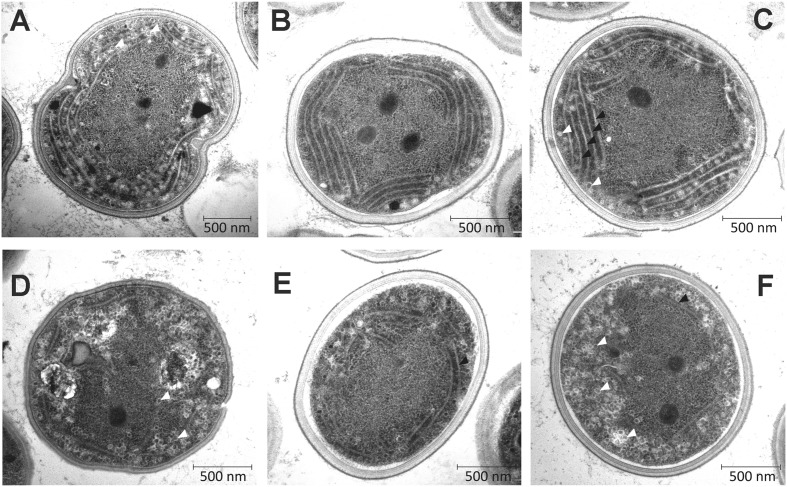
**Transmission electron micrographs of WT and Δ*ycf54* cells.** Ultrathin sections from *Synechocystis* WT **(A–C)** and Δ*ycf54* cells **(D–F)** grown photomixotrophically under low light conditions. White arrows indicate thylakoid membranes and white triangles indicate glycogen granules.

In order to investigate the effects of removal of 87% of the cellular Chl on the ultrastructure of Δ*ycf54* cells, electron microscopy of negatively stained thin cell sections was performed. Electron micrographs are shown in **Figure 8**. In the WT the thylakoids are observed as parallel stacks of two to five membranes that closely follow the contour of the cell membrane (**Figures 8A–C**), but no such organized thylakoid membranes are visible in micrographs of the Δ*ycf54* mutant (**Figures 8B–D**). Instead, membrane-like structures are dispersed throughout the cytoplasm of the cell. These results suggest key role of photosystems in the formation of the highly ordered thylakoid structures.

## Discussion

The MgPME-cyclase is the least understood component in the Chl biosynthetic pathway, and current knowledge of the individual components of the MgPME-cyclase had been limited to homologs of the *Rubrivivax gelatinosus* AcsF protein. Previous work identified two genes in *Synechocystis*, *sll1214*, and *sll1874*, as *acsF* homologs, which encode a membrane associated component of the MgPME-cyclase (Minamizaki et al., 2008; Peter et al., 2009). AcsF and its homologs contain a putative di-iron site and are thus viewed as the true catalytic subunit of the MgPME-cyclase (Tottey et al., 2003). The discovery of another gene, *ycf54*, that plays an important role in cyclase activity (Albus et al., 2012; Hollingshead et al., 2012) showed that other components are required, but so far it is not possible to assign a catalytic or assembly-related function to the Ycf54 protein. Further work on the role of Ycf54 required a fully segregated Δ*ycf54* mutant, which is reported herein.

In our efforts to construct a fully segregated Δ*ycf54* mutant, we discovered it was only possible to completely delete the *ycf54* gene in one specific substrain of *Synechocystis* (GT-W). One possible explanation for this finding was elucidated by analysis of the GT-W genome, which contains a long (~100 kbp) chromosomal duplication that covers one hundred genes, including *cycI (sll1214*; Bečková et al., submitted). This chromosomal duplication is not present in any of the other *Synechocystis* substrains for which a genome sequence is available (Kanesaki et al., 2012; Trautmann et al., 2012). Given the duplication of *cycI* in GT-W, it is likely that *cycI* expression is increased in this strain. A very low level of CycI is a hallmark of the strains in which we partially or completely inactivated the *ycf54* gene. Thus, we hypothesize that the doubled expression of the *sll1214* gene coding for CycI may suppress the lethality of inactivating the *ycf54* gene. This hypothesis is in agreement with our observation that in the absence of Ycf54 the CycI cyclase component is destabilized (**Figures 4** and **5**) and the remaining CycI content is probably very close to a threshold essential for viability.

Analysis of the pigments that accumulate in the Δ*ycf54* mutant could provide clues regarding the role of the Ycf54 protein, and a previous analysis showed that the partially segregated *ycf54* mutant accumulates MgPME, the substrate of the cyclase. In addition, there was an unknown pigment (**Figure 2**) that was suggested to be an intermediate in the cyclase reaction (Hollingshead et al., 2012), on the basis that the 433 nm Soret absorbance peak falls between the Soret peaks of the cyclase substrate MgPME (416 nm) and the PChlide product (440 nm). Similarly, early work with greening cucumber cotyledons had found pigments with emission maxima between 434 and 436 nm, proposed to be biosynthetic intermediates between MgPME and PChlide (Rebeiz et al., 1975). Identification of the unknown pigment as Mg-3-formyl-PME suggests that this pigment is not an intermediate in the cyclase reaction, as it is highly unlikely that this would produce a Chl pigment modified at the C3 position. Rather, Mg-3-formyl-PME bears a striking resemblance to Chl *d*, the major light harvesting pigment found in the cyanobacterium *Acaryochloris marina* (Miyashita et al., 1996). The pathway and reaction mechanism of Chl *d* biosynthesis in *Acaryochloris marina* have not yet been elucidated but, based on the genome sequence, Chl *d* is thought to be derived from Chl *a* (Swingley et al., 2008). Previously, Chl *d* has been synthesized in low yields in aqueous acetone from Chl *a* by treatment with papain (Koizumi et al., 2005) and peroxide (Aoki et al., 2011) and in much higher yields from Chl *a* incubated with thiophenol and acetic acid in tetrahydrofuran (Fukusumi et al., 2012). Given the high accumulation of MgPME in Δ*ycf54*, it is likely that reactive oxygen species, including peroxide, convert the MgPME 3-vinyl group, leading to the formation of Mg-3-formyl-PME.

The identification of Mg-3-formyl-PME as an oxidation product of the substrate rather than a catalytic intermediate is not consistent with a catalytic role for Ycf54 in the MgPME-cyclase complex. However, the hypothesis by Hollingshead et al. (2012) that Ycf54 plays a role in the assembly or stabilization of the catalytic MgPME-cyclase enzyme complex remains valid. By using FLAG-CycI as bait in pulldown assays, combined with quantitative MS analysis, we demonstrated that the absence of Ycf54 affects formation of a complex between CycI and enzymes further down the pathway (POR, DVR, and ChlP). In particular, the almost complete absence of DVR in the pulldown from the Δ*ycf54* strain provides a strong evidence that Ycf54 facilitates formation of such a complex; in contrast to POR and ChlP the stability of DVR in the mutant does not seem to be compromised and thus this result cannot be explained by a hypothetical fast degradation of this enzyme during pulldown assay. Indeed, the present data also provide evidence for an interaction between Cycl1 and POR, DVR, and ChlP, which aligns well with data obtained by Kauss et al. (2012) who performed pulldown experiments with the *Arabidopsis* FLU protein. These analyses found that FLU forms a complex with CHL27, the *Arabidopsis* AcsF homolog, PORB, PORC, and ChlP. In *Synechocystis* at least, it appears that Ycf54 plays no direct catalytic role, and that it is important for the formation and maintenance of a Chl biosynthetic complex, with disruption of this complex possibly triggering degradation of CycI and consequently Chl deficiency. However, a wider role for Ycf54 in governing the whole pathway appears to be excluded by the lack of effect of the Δ*ycf54* on components of the ChlG-HliD-Ycf39 complex that operates at the end of the pathway. This complex likely coordinates Chl delivery to the membrane-intrinsic apparatus for insertion and translocation of apoproteins of the photosynthetic apparatus (Chidgey et al., 2014). It is notable that although the CycI is almost exclusively associated with the membrane fraction under moderate light conditions Ycf54 is distributed equally between membrane and soluble fractions (**Figure 4B**). It is not known whether membrane-bound or soluble Ycf54 is critical for the CycI stability but there is a possibility that dissociation of Ycf54 from a membrane-bound assembly of CycI, POR, and DVR enzymes triggers degradation of CycI. Such a mechanism might regulate CycI activity at post-translational level and allow the cell to respond quickly to fluctuations in the environment.

Deletion of the *ycf54* gene generated a *Synechocystis* strain with very low levels of Chl, facilitating our studies on the cellular effects of a greatly lowered flux down the Chl biosynthetic pathway. It has long been known that photosystem biosynthesis requires Chl (Mullet et al., 1990; Eichacker et al., 1992; Müller and Eichacker, 1999), so we took advantage of the low Chl levels in the Δ*ycf54* mutant to investigate the effects of Chl depletion on the synthesis and assembly of the photosystems. Although the levels of some Chl biosynthesis enzymes are altered in the Δ*ycf54* mutant the ChlG-HliD-Ycf39 complex is unaffected, allowing the effects of reduced flux down the Chl pathway on Chl binding proteins to be investigated without disrupting ChlG-HliD-Ycf39 interactions with the YidC/Alb3 insertase and the consequent synthesis of nascent photosystem polypeptides. In addition, we were able to investigate the accumulation of “minor” Chl binding proteins, including cytochrome *b*_6_*f* (Kurisu et al., 2003), the Hli proteins (Staleva et al., 2015) and the Chl-synthase complex (Chidgey et al., 2014). Our findings show that whilst the Chl binding proteins PsaA/B and CP47 are highly sensitive to cellular Chl levels, the accumulation of CP43 and “minor” Chl binding proteins, including cytochrome *b*_6_*f*, is robust under Chl limiting conditions.

PSI is the main sink for *de novo* Chl (Kopečná et al., 2013); our results (**Figure 7**) show that synthesis of the core PSI subunits PsaA/B is impaired in the absence of Ycf54, i.e., under *de novo* Chl limiting conditions, suggesting that *Synechocystis* is unable to recycle Chl molecules released from degraded complexes for the synthesis of new PSI complexes. In comparison, the dependence of PSII biogenesis on the availability of *de novo* Chl modules appears to be more complex, as previous studies show Chl molecules are re-cycled during PSII synthesis and repair (Kopečná et al., 2013, 2015) via re-phytylation of chlorophyllide (Vavilin and Vermaas, 2007). The PSII complex assembles in a modular fashion, starting with the association of D1 and D2 assembly modules, to form the RCII* complex. This is followed by attachment of a CP47 module, then a CP43 module, then the lumenal extrinsic proteins and the oxygen-evolving Mn_4_CaO_5_ complex (reviewed in Komenda et al., 2012). Despite the large decrease in cellular Chl levels in Δ*ycf54*, all components of the RCII* complex are synthesized in adequate amounts and assembled. We hypothesize that synthesis of the RCII* complex is enabled by the continuous recycling of a relatively stable pool of Chl molecules made available during the RCII* assembly/degradation cycle. Evidence that RCII* contains Chl as well pheophytin, carotenoids and heme cofactors has been shown previously (Knoppová et al., 2014). We cannot exclude the possibility that in the Δ*ycf54* mutant there is a pool of the RCII* complex that lacks Chl. However, we did not observe any shift of electrophoretic mobility even for the ^35^S labeled RCII* that would indicate presence of a hypothetical Chl-less RCII*. Furthermore, Δ*ycf54* contains some functional PSII complexes, which requires that at least some RCII* with cofactors has to be synthesized en route to the fully assembled PSII.

Our findings also show that CP43 can accumulate as an unassembled module in Δ*ycf54* even though the synthesis is very limited (**Figures 6A** and **7**). In contrast, CP47 seems to be unstable in Δ*ycf54*, which suggests that CP47 is the *de novo* Chl sensitive component of PSII biogenesis. This observation is consistent with previous work on the accumulation of PSII subunits in Δ*por* (Kopečná et al., 2013) and Δ*gun4* (Sobotka et al., 2008) mutants, disrupted in the PChlide reduction and Mg-chelatase steps, respectively. As also seen for Δ*ycf54*, the Δ*por* and Δ*gun4* strains accumulate the PSII core complex RCII* and the PSII antenna CP43, but CP47 synthesis is not observed (Sobotka et al., 2008; Kopečná et al., 2013). It is not currently known why CP47 is more sensitive to the availability of *de novo* Chl than the similar CP43 subunit, although it has been recently observed that the newly synthesized CP43, but not CP47, subunit is attached to a PSI complex (Kopečná et al., 2015). We tentatively speculate that the situation in the mutant leads frequently to the synthesis of aberrant CP47 lacking one or more Chl molecules. The synthesis of CP43 might be less error-prone because Chl molecules bound to the periphery of PSI could be used for the assembly of this complex.

In summary, the role of Ycf54 in the MgPME-cyclase complex has been elucidated further. This work shows that whilst Ycf54 is required for stabilization of Cyc1, the known catalytic component of the MgPME-cyclase, the protein itself is unlikely to play a key catalytic role in the formation of the fifth isocyclic ring. Furthermore, Ycf54 does not appear to be directly implicated in Chl phytolation or Chl insertion into proteins. The construction of a Δ*ycf54* mutant has provided a useful tool to investigate the effects of reduced *de novo* Chl on the biosynthesis of cyanobacterial Chl binding proteins, highlighting the differing requirements for Chl exhibited by proteins within the PSI and PSII light harvesting complexes that bind this pigment. Insights into the catalytic cycle of the MgPME-cyclase remain elusive and further work is required to determine the exact molecular mechanisms of this enzyme.

## Author Contributions

SH, JK, DA, LB, PJ, and GC performed the research; MD, MW, RS, and CNH designed the experiments, and SH, DA, PJ, MD, MW, RS, and CNH wrote the paper.

## Conflict of Interest Statement

The authors declare that the research was conducted in the absence of any commercial or financial relationships that could be construed as a potential conflict of interest.

## Footnotes

1 www.proteowizard.sourceforge.net

2 www.uniprot.org/proteomes/UP000001425

3 http://proteomecentral.proteomexchange.org

## References

[B1] Albus C. A. Salinas A. Czarnecki O. Kahlau S. Rothbart M. Thiele W. (2012). LCAA, a novel factor required for magnesium protoporphyrin monomethylester cyclase accumulation and feedback control of aminolevulinic acid biosynthesis in Tobacco. *Plant Physiol.* 160 1923–1939. 10.1104/pp.112.206045 23085838 PMC3510121

[B2] Aoki K. Itoh S. Furukawa H. Nakazato M. Iwamoto K. Shiraiwa Y. (2011). “Enzymatic and non-enzymatic conversion of Chl a to Chl d,” in *Proceedings of the 5th Asia and Oceania Conference on Photobiology* Nara.

[B3] Boehm M. Romero E. Reisinger V. Yu J. Komenda J. Eichacker L. A. (2011). Investigating the early stages of Photosystem II assembly in *Synechocystis* sp PCC 6803: isolation of CP47 and CP43 complexes. *J. Biol. Chem.* 286 14812–14819. 10.1074/jbc.M110.207944 21339295 PMC3083219

[B4] Boehm M. Yu J. Reisinger V. Bečková M. Eichacker L. A. Schlodder E. (2012). Subunit composition of CP43-less photosystem II complexes of *Synechocystis* sp PCC 6803: implications for the assembly and repair of photosystem II. *Philos. Trans. R. Soc. B Biol. Sci.* 367 3444–3454. 10.1098/rstb.2012.0066 PMC3497071 23148271

[B5] Boldareva-Nuianzina E. N. Bláhová Z. Sobotka R. Koblížek M. (2013). Distribution and origin of oxygen-dependent and oxygen-independent forms of Mg-protoporphyrin monomethylester cyclase among phototrophic *proteobacteria*. *Appl. Environ. Microbiol.* 79 2596–2604. 10.1128/AEM.00104-13 23396335 PMC3623192

[B6] Bollivar D. Braumann I. Berendt K. Gough S. P. Hansson M. (2014). The Ycf54 protein is part of the membrane component of Mg-protoporphyrin IX monomethyl ester cyclase from barley (*Hordeum vulgare* L.). *FEBS J.* 281 2377–2386. 10.1111/febs.12790 24661504

[B7] Canniffe D. P. Jackson P. J. Hollingshead S. Dickman M. J. Hunter C. N. (2013). Identification of an 8-vinyl reductase involved in bacteriochlorophyll biosynthesis in *Rhodobacter sphaeroides* and evidence for the existence of a third distinct class of the enzyme. *Biochem. J.* 450 397–405. 10.1042/BJ20121723 23252506

[B8] Chidgey J. W. Linhartová M. Komenda J. Jackson P. J. Dickman M. J. Canniffe D. P. (2014). A cyanobacterial chlorophyll synthase-HliD complex associates with the Ycf39 protein and the YidC/Alb3 insertase. *Plant Cell* 26 1267–1279. 10.1105/tpc.114.124495 24681617 PMC4001383

[B9] Chua N. H. Blobel G. Siekevitz P. Palade G. E. (1976). Periodic variations in the ratio of free to thylakoid-bound chloroplast ribosomes during the cell cycle of *Chlamydomonas reinhardtii*. *J. Cell Biol.* 71 497–514. 10.1083/jcb.71.2.497 993261 PMC2109746

[B10] Dobáková M. Sobotka R. Tichý M. Komenda J. (2009). Psb28 protein is involved in the biogenesis of the photosystem II inner antenna CP47 (PsbB) in the cyanobacterium *Synechocystis* sp. PCC 6803. *Plant Physiol.* 149 1076–1086. 10.1104/pp.108.130039 19036835 PMC2633855

[B11] Eichacker L. Paulsen H. Rüdiger W. (1992). Synthesis of chlorophyll a regulates translation of chlorophyll a apoproteins P700, CP47, CP43 and D2 in barley etioplasts. *Eur. J. Biochem.* 205 17–24. 10.1111/j.1432-1033.1992.tb16747.x 1555577

[B12] Eichacker L. A. Helfrich M. Rüdiger W. Muller B. (1996). Stabilization of chlorophyll a-binding apoproteins P700, CP47, CP43, D2, and D1 by chlorophyll a or Zn-pheophytin a. *J. Biol. Chem.* 271 32174–32179. 10.1074/jbc.271.50.32174 8943272

[B13] Fukusumi T. Matsuda K. Mizoguchi T. Miyatake T. Ito S. Ikeda T. (2012). Non-enzymatic conversion of chlorophyll-a into chlorophyll-d in vitro: a model oxidation pathway for chlorophyll-d biosynthesis. *FEBS Lett.* 586 2338–2341. 10.1016/j.febslet.2012.05.036 22659188

[B14] Hollingshead S. Kopečná J. Jackson P. J. Canniffe D. P. Davison P. A. Dickman M. J. (2012). Conserved chloroplast open-reading frame ycf54 is required for activity of the magnesium protoporphyrin monomethylester oxidative cyclase in *Synechocystis* PCC 6803. *J. Biol. Chem.* 287 27823–27833. 10.1074/jbc.M112.352526 22711541 PMC3431679

[B15] Jordan P. Fromme P. Witt H. T. Klukas O. Saenger W. Krauss N. (2001). Three-dimensional structure of cyanobacterial photosystem I at 2.5 Å resolution. *Nature* 411 909–917. 10.1038/35082000 11418848

[B16] Kalb V. F. Bernlohr R. W. (1977). A new spectrophotometric assay for protein in cell extracts. *Anal. Biochem.* 82 362–371. 10.1016/0003-2697(77)90173-7 20815

[B17] Kanesaki Y. Shiwa Y. Tajima N. Suzuki M. Watanabe S. Sato N. (2012). Identification of substrain-specific mutations by massively parallel whole-genome resequencing of *Synechocystis* sp. PCC 6803. *DNA Res.* 19 67–79. 10.1093/dnares/dsr042 22193367 PMC3276265

[B18] Kauss D. Bischof S. Steiner S. Apel K. Meskauskiene R. (2012). FLU, a negative feedback regulator of tetrapyrrole biosynthesis, is physically linked to the final steps of the Mg++-branch of this pathway. *FEBS Lett.* 586 211–216. 10.1016/j.febslet.2011.12.029 22212719

[B19] Ke S. H. Madison E. L. (1997). Rapid and efficient site-directed mutagenesis by single-tube ‘megaprimer’ PCR method. *Nucleic Acids Res.* 25 3371–3372. 10.1093/nar/25.16.3371 9241254 PMC146891

[B20] Knoppová J. Sobotka R. Tichý M. Yu J. Koník P. Halada P. (2014). Discovery of a chlorophyll binding protein complex involved in the early steps of photosystem II assembly in *Synechocystis*. *Plant Cell* 26 1200–1212. 10.1105/tpc.114.123919 24681620 PMC4001378

[B21] Koizumi H. Itoh Y. Hosoda S. Akiyama M. Hoshino T. Shiraiwa Y. (2005). Serendipitous discovery of Chl d formation from Chl a withpapain. *Sci. Technol. Adv. Mater.* 6 551–557. 10.1016/j.stam.2005.06.022

[B22] Komenda J. Nickelsen J. Tichý M. Prášil O. Eichacker L. A. Nixon P. J. (2008). The cyanobacterial homologue of HCF136/YCF48 is a component of an early photosystem II assembly complex and is important for both the efficient assembly and repair of photosystem II in *Synechocystis* sp PCC 6803. *J. Biol. Chem.* 283 22390–22399. 10.1074/jbc.M801917200 18550538

[B23] Komenda J. Reisinger V. Müller B. C. Dobáková M. Granvogl B. Eichacker L. A. (2004). Accumulation of the D2 protein is a key regulatory step for assembly of the photosystem II reaction center complex in *Synechocystis* PCC 6803. *J. Biol. Chem.* 279 48620–48629. 10.1074/jbc.M405725200 15347679

[B24] Komenda J. Sobotka R. Nixon P. J. (2012). Assembling and maintaining the Photosystem II complex in chloroplasts and cyanobacteria. *Curr. Opin. Plant Biol.* 15 245–251. 10.1016/j.pbi.2012.01.017 22386092

[B25] Kopečná J. Pilný J. Krynická V. Tomèala A. Kis M. Gombos Z. (2015). Lack of phosphatidylglycerol inhibits chlorophyll biosynthesis at multiple sites and limits chlorophyllide reutilization in *Synechocystis* sp. Strain PCC 6803. *Plant Physiol.* 169 1307–1317. 10.1104/pp.15.01150 26269547 PMC4587476

[B26] Kopečná J. Sobotka R. Komenda J. (2013). Inhibition of chlorophyll biosynthesis at the protochlorophyllide reduction step results in the parallel depletion of Photosystem I and Photosystem II in the cyanobacterium *Synechocystis* PCC 6803. *Planta* 237 497–508. 10.1007/s00425-012-1761-4 23011568

[B27] Kurisu G. Zhang H. M. Smith J. L. Cramer W. A. (2003). Structure of the cytochrome b6f complex of oxygenic photosynthesis: tuning the cavity. *Science* 302 1009–1014. 10.1126/science.1090165 14526088

[B28] Minamizaki K. Mizoguchi T. Goto T. Tamiaki H. Fujita Y. (2008). Identification of two homologous genes, chlAI and chlAII, that are differentially involved in isocyclic ring formation of chlorophyll a in the cyanobacterium *Synechocystis* sp PCC 6803. *J. Biol. Chem.* 283 2684–2692. 10.1074/jbc.M708954200 18039649

[B29] Miyashita H. Ikemoto H. Kurano N. Adachi K. Chihara M. Miyachi S. (1996). Chlorophyll d as a major pigment. *Nature* 383 402 10.1038/383402a0

[B30] Müller B. Eichacker L. A. (1999). Assembly of the D1 precursor in monomeric photosystem II reaction center precomplexes precedes chlorophyll a-triggered accumulation of reaction center II in barley etioplasts. *Plant Cell* 11 2365–2377. 10.2307/3870961 10590164 PMC144137

[B31] Mullet J. E. Klein P. G. Klein R. R. (1990). Chlorophyll regulates accumulation of the plastid encoded chlorophyll apoprotein CP43 and apoprotein D1 by increasing apoprotein stability. *Proc. Natl. Acad. Sci. U.S.A.* 87 4038–4042. 10.1073/pnas.87.11.4038 2349216 PMC54042

[B32] Nixon P. J. Michoux F. Yu J. Boehm M. Komenda J. (2010). Recent advances in understanding the assembly and repair of photosystem II. *Ann. Bot.* 106 1–16. 10.1093/aob/mcq059 20338950 PMC2889791

[B33] Peter E. Salinas A. Wallner T. Jeske D. Dienst D. Wilde A. (2009). Differential requirement of two homologous proteins encoded by sll1214 and sll1874 for the reaction of Mg protoporphyrin monomethylester oxidative cyclase under aerobic and micro-oxic growth conditions. *Biochim. Biophys. Acta* 1787 1458–1467. 10.1016/j.bbabio.2009.06.006 19540827

[B34] Pinta V. Picaud M. Reiss-Husson F. Astier C. (2002). *Rubrivivax gelatinosus* acsF (previously orf358) codes for a conserved, putative binuclear-iron-cluster-containing protein involved in aerobic oxidative cyclization of Mg-protoporphyrin IX monomethylester. *J. Bacteriol.* 184 746–753. 10.1128/JB.184.3.746-753.2002 11790744 PMC139524

[B35] Porra R. Thompson W. Kriedemann P. (1989). Determination of accurate extinction coefficients and simulataneous equations for assaying chlorophylls a and b extracted with four different solvents: verification of the concentration of chlorophyll standards by atomic absorption spectroscopy. *Biochim. Biophys. Acta* 975 384–389. 10.1016/S0005-2728(89)80347-0

[B36] Porra R. J. Schafer W. Gadon N. Katheder I. Drews G. Scheer H. (1996). Origin of the two carbonyl oxygens of bacteriochlorophyll alpha – Demonstration of two different pathways for the formation of ring E in *Rhodobacter sphaeroides* and *Roseobacter denitrificans*, and a common hydratase mechanism for 3-acetyl group formation. *Eur. J. Biochem.* 239 85–92. 10.1111/j.1432-1033.1996.0085u.x 8706723

[B37] Promnares K. Komenda J. Bumba L. Nebesarova J. Vacha F. Tichy M. (2006). Cyanobacterial small chlorophyll-binding protein ScpD (HliB) is located on the periphery of photosystem II in the vicinity of PsbH and CP47 subunits. *J. Biol. Chem.* 281 32705–32713. 10.1074/jbc.M606360200 16923804

[B38] Rebeiz C. A. Mattheis J. R. Smith B. B. Rebeiz C. Dayton D. F. (1975). Chloroplast biogenesis. Biosynthesis and accumulation of Mg-protoprophyrin IX monoester and longer wavelength metalloporphyrins by greening cotyledons. *Arch. Biochem. Biophys.* 166 446–465. 10.1016/0003-9861(75)90408-7 1119802

[B39] Rippka R. Deruelles J. Waterbury J. Herdman M. Stanier R. (1979). Generic assignments, strain histories and properties of pure cultures of cyanobacteria. *Microbiology* 111 1–61. 10.1099/00221287-111-1-1

[B40] Sobotka R. Duerhring U. Komenda J. Peter E. Gardian Z. Tichy M. (2008). Importance of the cyanobacterial GUN4 protein for chlorophyll metabolism and assembly of photosynthetic complexes. *J. Biol. Chem.* 283 25794–25802. 10.1074/jbc.M803787200 18625715 PMC3258849

[B41] Sobotka R. Tichy M. Wilde A. Hunter C. N. (2011). Functional assignments for the carboxyl-terminal domains of the ferrochelatase from *Synechocystis* PCC 6803: the CAB domain plays a regulatory role, and region II is essential for catalysis. *Plant Physiol.* 155 1735–1747. 10.1104/pp.110.167528 21081693 PMC3091120

[B42] Staleva H. Komenda J. Shukla M. K. Šlouf V. Kaòa R. Polívka T. (2015). Mechanism of photoprotection in the cyanobacterial ancestor of plant antenna proteins. *Nat. Chem. Biol.* 11 287–291. 10.1038/nchembio.1755 25706339

[B43] Stott K. Stonehouse J. Keeler J. Hwang T. Shaka A. (1995). Excitation sculpting in high-resolution nuclear magnetic resonance spectroscopy: application to selective NOE experiments. *J. Am. Chem. Soc.* 117 4199–4200. 10.1021/ja00119a048

[B44] Swingley W. D. Chen M. Cheung P. C. Conrad A. L. Dejesa L. C. Hao J. (2008). Niche adaptation and genome expansion in the chlorophyll d-producing cyanobacterium *Acaryochloris marina*. *Proc. Natl. Acad. Sci. U.S.A.* 105 2005–2010. 10.1073/pnas.0709772105 18252824 PMC2538872

[B45] Tottey S. Block M. A. Allen M. Westergren T. Albrieux C. Scheller H. V. (2003). *Arabidopsis* CHL27 located in both envelope and thylakoid membranes, is required for the synthesis of protochlorophyllide. *Proc. Natl. Acad. Sci. U.S.A.* 100 16119–16124. 10.1073/pnas.2136793100 14673103 PMC307702

[B46] Trautmann D. Voss B. Wilde A. Al-Babili S. Hess W. R. (2012). Microevolution in cyanobacteria: re-sequencing a motile substrain of *Synechocystis* sp. PCC 6803. *DNA Res.* 19 435–448. 10.1093/dnares/dss024 23069868 PMC3514855

[B47] Umena Y. Kawakami K. Shen J. R. Kamiya N. (2011). Crystal structure of oxygen-evolving photosystem II at a resolution of 1.9 Å. *Nature* 473 55–60. 10.1038/nature09913 21499260

[B48] van de Meene A. M. Hohmann-Marriott M. F. Vermaas W. F. Roberson R. W. (2006). The three-dimensional structure of the cyanobacterium *Synechocystis* sp. PCC 6803. *Arch. Microbiol.* 184 259–270. 16320037 10.1007/s00203-005-0027-y

[B49] Vavilin D. Vermaas W. (2007). Continuous chlorophyll degradation accompanied by chlorophyllide and phytol reutilization for chlorophyll synthesis in *Synechocystis* sp PCC 6803. *Biochim. Biophys. Acta* 1767 920–929. 10.1016/j.bbabio.2007.03.010 17499209

[B50] Wilde A. Mikolajczyk S. Alawady A. Lokstein H. Grimm B. (2004). The gun4 gene is essential for cyanobacterial porphyrin metabolism. *FEBS Lett.* 571 119–123. 10.1016/j.febslet.2004.06.063 15280028

[B51] Zhang H. Liu H. Blankenship R. E. Gross M. L. (2015). Isotope-encoded carboxyl group footprinting for mass spectrometry-based protein conformational studies. *J. Am. Soc. Mass Spectrom.* 27 178–181. 10.1007/s13361-015-1260-5 26384685 PMC4688080

[B52] Zouni A. Witt H. T. Kern J. Fromme P. Krauss N. Saenger W. (2001). Crystal structure of photosystem II from *Synechococcus elongatus* at 3.8 Å resolution. *Nature* 409 739–743. 10.1038/35055589 11217865

