# Supplementary material for: Synthesis of Chlorophyll-Binding Proteins in a Fully Segregated Δycf54 Strain of the Cyanobacterium Synechocystis PCC 6803
Source: Front Plant Sci. 2016 Mar 17;7:292. doi: 10.3389/fpls.2016.00292 (PMC4794507; doi:10.3389/fpls.2016.00292)
Supplement: Supplementary file 1 [file Data_Sheet_1.PDF]

Supplementary Material

Synthesis of chlorophyll-binding proteins in a fully-segregated Δ*ycf54* strain of the cyanobacterium *Synechocystis* PCC 6803

Sarah Hollingshead[1,5], Jana Kopečná[2], David R Armstrong[1], Lenka Bučinská[2,3], Philip J Jackson[1,4], Guangyu E. Chen[1], Mark J Dickman[4], Michael P. Williamson[1], Roman Sobotka[2,3] , C. Neil Hunter[1*]

*** Correspondence:** Prof. C. Neil Hunter, Department of Molecular Biology and Biotechnology, University of Sheffield, Firth Court, Western Bank, Sheffield S10 2TN, United Kingdom

1. Supplementary Figures

NMR data for identification of 3-formyl MgPME

All spectra were acquired on a Bruker Avance DRX at 600 MHz at 298 K, using a cryoprobe. The sample was in a sealed 5 mm tube. Peaks were assigned by comparison to protochlorophyllide, and using TOCSY, ^{13}C HSQC and one-dimensional NOE spectra. The TOCSY spectrum is shown in Fig. S1.

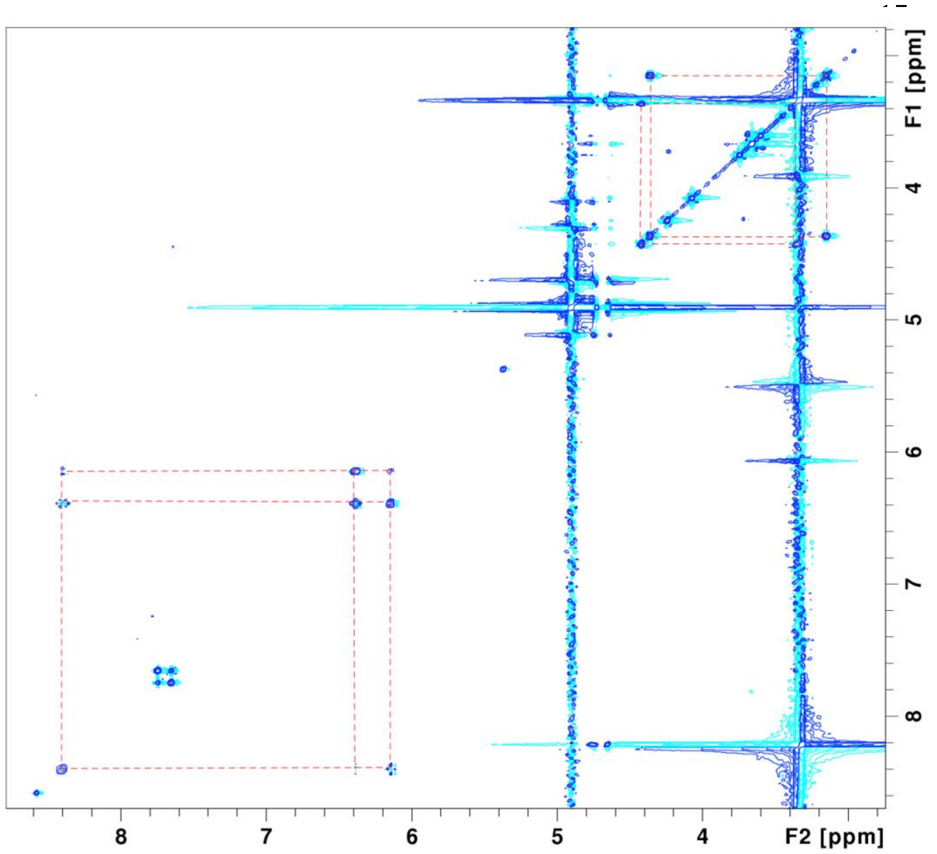

Supplementary Figure S1. 2D ^1H TOCSY spectrum of 3-formyl MgPME. The spectrum was acquired using spectral widths of 12.5 ppm in both dimensions, with 1k complex points in the direct dimension and 512 complex points in the indirect dimension. The spin lock was 45 ms, using a DIPSI pulse sequence with a power of 8.3 kHz. The two intense peaks at 4.9 and 3.3 ppm come from water and methanol respectively.

There are only three *J*-coupling systems in the molecule, indicated by red lines in the spectrum, originating from the 8-vinyl sidechain (6-8 ppm) and the two propionate sidechains, which are partially overlapped by the methanol signal.

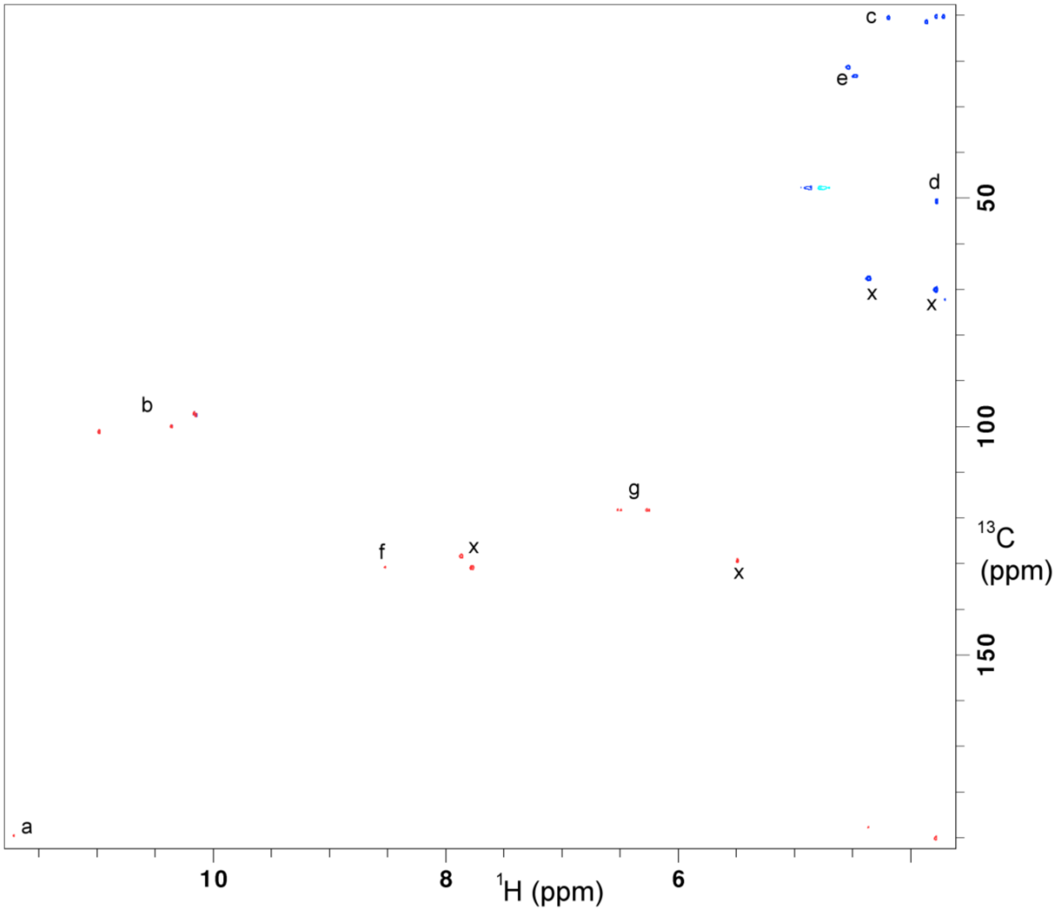

Supplementary Figure S2. Superimposed natural abundance ^{13}C HSQC spectra of 3-formyl MgPME, with carbon offsets of 60 ppm (blue) and 140 ppm (red). The spectra used echo-antiecho gradient selection, with 1k x 100 complex points (^{1}H x ^{13}C) and 256 scans per increment. In order to cover the carbon spectral width effectively, two spectra were acquired with different carbon offsets, of 60 and 140 ppm, and ^{13}C spectral widths of 120 ppm. The two spectra are overlaid in Fig S2, with the 60 ppm offset in blue and the 140 ppm offset superimposed, in red. The signals from the four *meso* groups are indicated by *b*, and are present in both spectra.

Signals of interest are marked on the spectrum: *a*, formyl group; *b*, *meso* groups; *c*, C-methyls; *d*, propionate O-methyl; *e*, propionate sidechains; *f* and *g*, 8-vinyl group; *x*, solvent and impurities. The two peaks at bottom right are folded in to the red spectrum from 70 ppm.

The assignment was completed by means of one-dimensional gradient-selected NOEs, using a double pulsed field gradient spin echo selective experiment (Stott et al, 1995). A key element of the pulse sequence is the selective 180˚ pulse, which was an 80 ms Gaussian. The selectivity was very good, as evidenced by comparing the irradiations at the *meso* positions 10 and 15 in Fig S3, which are only 0.01 ppm apart yet have quite different NOE patterns. A further indicator of the quality of the spectra comes from the irradiations of impurities (indicated by * at the left of the spectrum), where the only NOEs seen are to solvent (and between the two impurity peaks at 7.8 ppm). An interesting feature of these spectra is that because of the high selectivity of the irradiation, NOEs involving *J*-coupled signals have an unusual splitting pattern because of selective population transfer effects (SPT: Neuhaus and Williamson 2000).

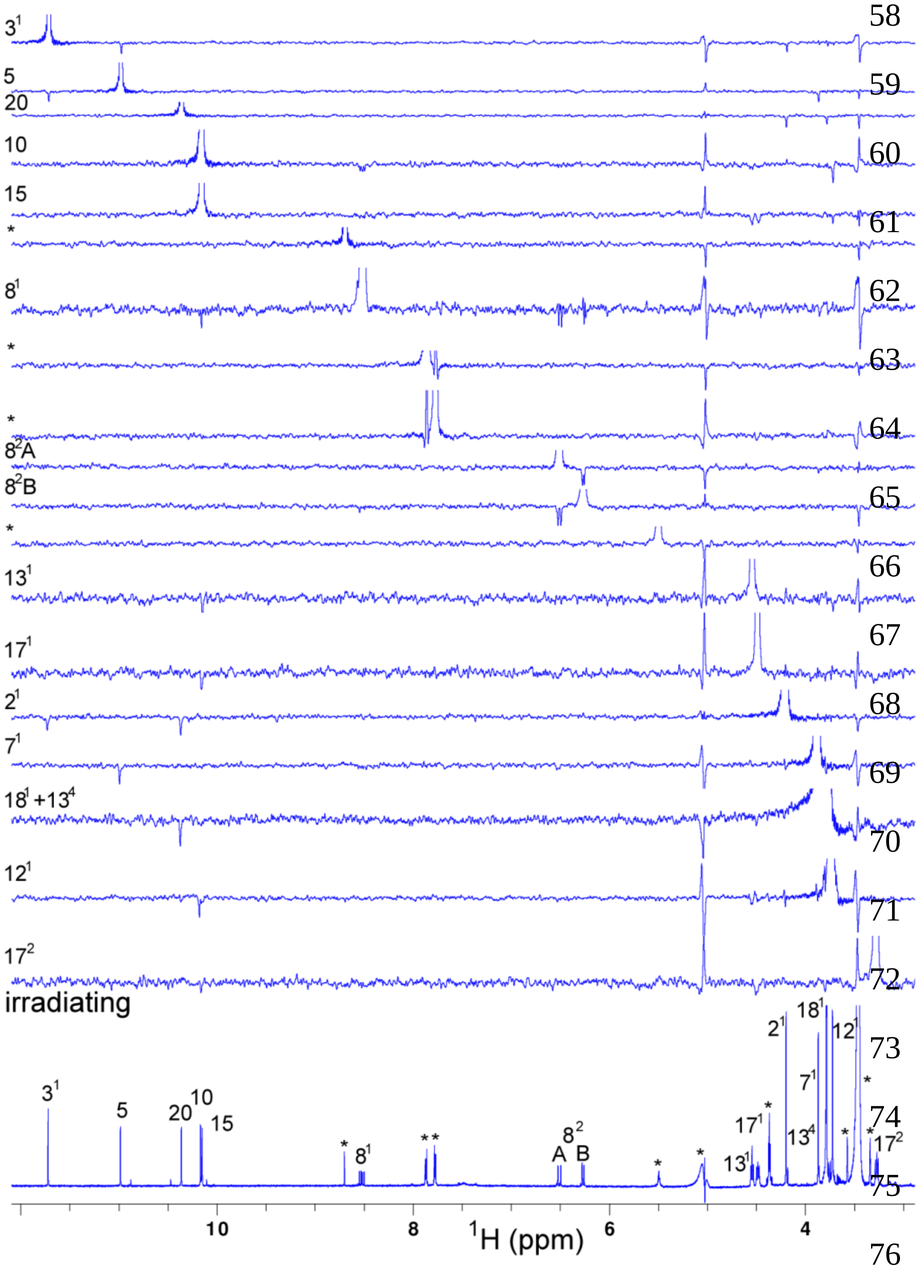

Supplementary Figure S3. One-dimensional double pulsed field gradient spin echo selective NOEs on 3-formyl MgPME. The reference spectrum is shown at the bottom: impurities and solvent are indicated by *. The experiment used the Bruker pulse program selnogp, with an 80 ms Gaussian 180˚ pulse, an NOE mixing time of 1 s, a relaxation delay of 3 s, and 2x1024 scans for each spectrum. The NOE and TOCSY connectivities observed in these spectra are summarised in Table S1 of the main supplementary information.

Neuhaus, D. and Williamson, M. P. (2000) *The Nuclear Overhauser Effect in Conformational Analysis*, Wiley-VCH, New York, Second Edition, Chapter 6.

Stott, K., Stonehouse, J., Keeler, J., Hwang, T., and Shaka, A. (1995). Excitation sculpting in high-resolution nuclear magnetic resonance spectroscopy: application to selective NOE experiments. *J Am Chem Soc* 117**,** 4199-4200.

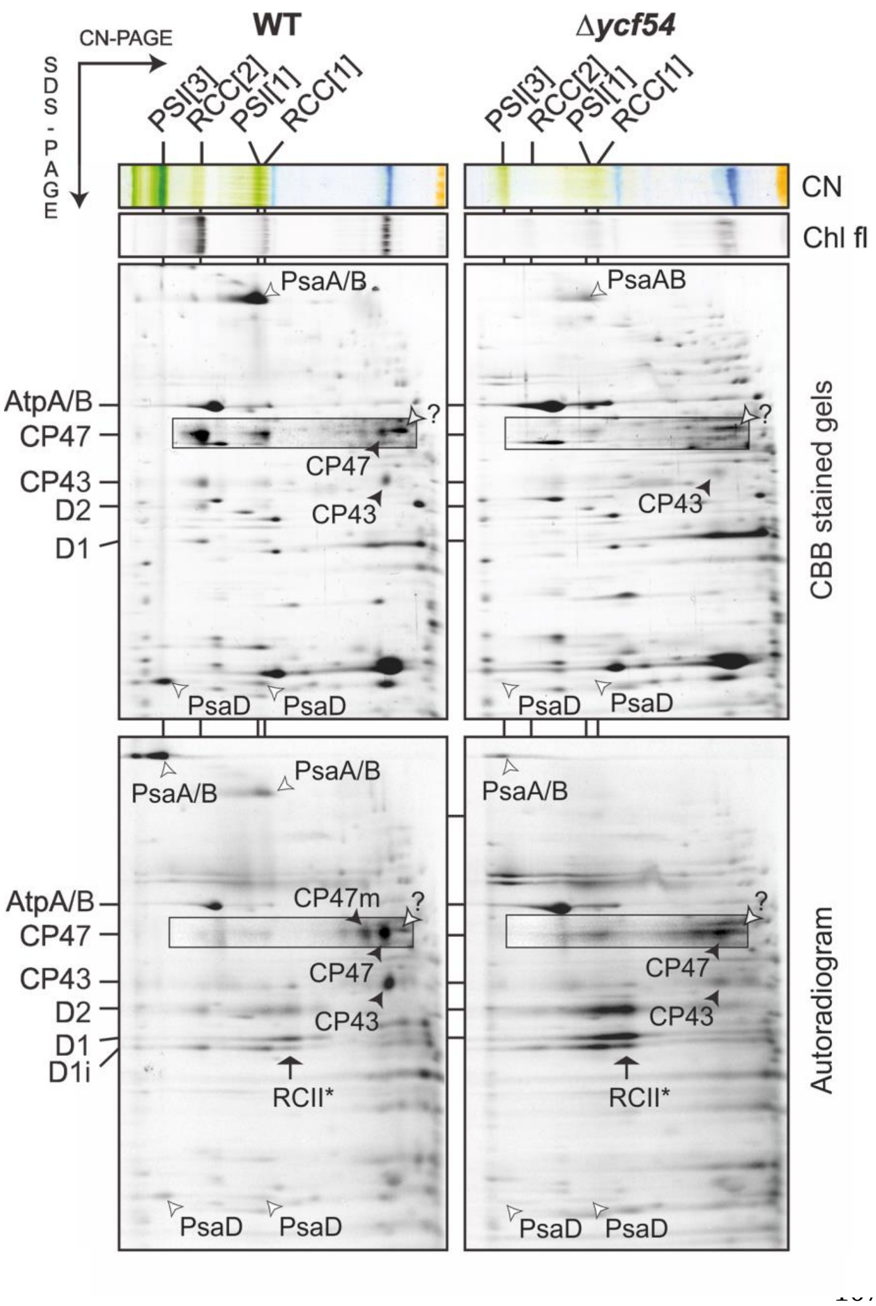

Supplementary Figure S4. Synthesis of the Chl-binding proteins in the Δ*ycf54* strain. Wild-type and mutant cells were radiolabeled with [^{35}S]Met/Cys mixture using a 30-min pulse. Isolated membrane proteins were separated by Clear-Native electrophoresis on a 4–14 % linear gradient gel, and another 12–20 % SDS-electrophoresis was used for the second dimension. For the **Δ***ycf54* membrane proteins isolated from three-times more cells were loading than for the control to obtain a detectable signal for weakly labelled proteins (PsaA/B). The 2D gels were stained with Coomassie Blue (upper panel) then dried, and the labelled proteins were then detected by a phosphoimager (lower panel). Protein complexes are designated as in Fig. 6. The inset box for each panel is overexposed to highlight CP47 and a neighbouring unknown protein (white arrowhead).

1.2 Supplementary Table 1. ^{1}H-NMR assignments with NOE and TOCSY correlations for 3-formyl MgPME

Proton	δ (ppm)	NOE cross signal with protons	TOCSY cross signal with protons
2^1	4.07	20, 3^1	-
3^1	11.60	2^1, 5	-
5	10.86	3^1, 7^1	-
7^1	3.74	5	-
8^1 (H_X)	8.40	8^2 (A,B), 10	8^2 (A,B)
8^2 (H_B, *trans*)	6.14	8^1, 8^2 (A)	8^1, 8^2 (A)
8^2 (H_A, *cis*)	6.38	7^1, 8^2 (B)	8^1, 8^2 (B)
10	10.04	8^1, 12^1	-
12^1	3.60	10	-
13^1	4.42	12^1, 15	13^2
13^2	3.32[a]	-	13^1
13^4	3.66[b]	-	-
15	10.03	13^1, 17^1	-
17^1	4.36	15, 17^2	17^2
17^2	3.15	17^1	17^1
18^1	3.66[b]	17^1, 20	-
20	10.24	2^1, 18^1	-

[a] – signal masked by methanol peak in 1D spectrum but visible in TOCSY spectrum.

[b] – 13^4 and 18^1 protons overlapped in 1D spectrum.
